# Cognitive function and mood at high altitude following acclimatization and use of supplemental oxygen and adaptive servoventilation sleep treatments

**DOI:** 10.1371/journal.pone.0217089

**Published:** 2019-06-12

**Authors:** Erica C. Heinrich, Matea A. Djokic, Dillon Gilbertson, Pamela N. DeYoung, Naa-Oye Bosompra, Lu Wu, Cecilia Anza-Ramirez, Jeremy E. Orr, Frank L. Powell, Atul Malhotra, Tatum S. Simonson

**Affiliations:** 1 Department of Medicine, Division of Pulmonary, Critical Care, and Sleep Medicine, University of California San Diego, La Jolla, California, United States of America; 2 Departamento de Ciencias Biológicas y Fisiológicas, Facultad de Ciencias y Filosofía, Universidad Peruana Cayetano Heredia, Lima, Peru; Medical University of Innsbruck, AUSTRIA

## Abstract

Impairments in cognitive function, mood, and sleep quality occur following ascent to high altitude. Low oxygen (hypoxia) and poor sleep quality are both linked to impaired cognitive performance, but their independent contributions at high altitude remain unknown. Adaptive servoventilation (ASV) improves sleep quality by stabilizing breathing and preventing central apneas without supplemental oxygen. We compared the efficacy of ASV and supplemental oxygen sleep treatments for improving daytime cognitive function and mood in high-altitude visitors (N = 18) during acclimatization to 3,800 m. Each night, subjects were randomly provided with ASV, supplemental oxygen (SpO_2_ > 95%), or no treatment. Each morning subjects completed a series of cognitive function tests and questionnaires to assess mood and multiple aspects of cognitive performance. We found that both ASV and supplemental oxygen (O_2_) improved daytime feelings of confusion (ASV: p < 0.01; O_2_: p < 0.05) and fatigue (ASV: p < 0.01; O_2_: p < 0.01) but did not improve other measures of cognitive performance at high altitude. However, performance improved on the trail making tests (TMT) A and B (p < 0.001), the balloon analog risk test (p < 0.0001), and the psychomotor vigilance test (p < 0.01) over the course of three days at altitude after controlling for effects of sleep treatments. Compared to sea level, subjects reported higher levels of confusion (p < 0.01) and performed worse on the TMT A (p < 0.05) and the emotion recognition test (p < 0.05) on nights when they received no treatment at high altitude. These results suggest that stabilizing breathing (ASV) or increasing oxygenation (supplemental oxygen) during sleep can reduce feelings of fatigue and confusion, but that daytime hypoxia may play a larger role in other cognitive impairments reported at high altitude. Furthermore, this study provides evidence that some aspects of cognition (executive control, risk inhibition, sustained attention) improve with acclimatization.

## Introduction

High-altitude exposure negatively impacts cognitive performance and mood. High-altitude migrants report increased anxiety, anger, and fatigue, and lower rates of positive mood and vigor than sea-level cohorts matched for age, sex, and education level [1]. Depression and suicide rates are also higher in high-altitude populations, even when accounting for socioeconomic factors [2,3]. This depressive mood, and additional cognitive impairment at high altitudes, may result from low oxygen availability [4–6] and/or poor sleep quality.

Low arterial oxygen pressure impairs central executive functions, perception/attention, and short-term memory [4,5,7]. Individuals exposed acutely to high altitude or simulated hypoxia demonstrate impaired complex reaction time [8–10], attention [11,12], memory [13,14], and perceptual-motor function [15], as well as an increase in irritability and depression [11,16]. Secondary to the initial hypoxic stimulus, increased ventilation in response to reduced arterial oxygen pressure results in systemic hypocapnia, which can decrease cerebral blood flow and exacerbate cognitive impairments [17]. Less is known about how cognition improves after acclimatization as cerebral spinal fluid pH and cerebral blood flow normalize [18].

Poor sleep quality is another likely contributor to negative mood and impaired cognition at high altitude, although this relationship has received less attention. Travelers to high altitude experience frequent arousals and low slow-wave sleep duration [11,19–21], which impair daytime cognitive function at sea level [22,23]. Poor sleep is also a symptom of acute mountain sickness (AMS), along with headache, gastrointestinal problems, fatigue, and lightheadedness [24]. These symptoms can contribute to negative dispositions reported in high-altitude visitors.

A common treatment for AMS and sleep disturbance at high altitude is supplemental oxygen. Nighttime supplemental oxygen decreases the apnea-hypopnea index (AHI), improves sleep quality [21,25,26], and can increase daytime saturation [27]. While oxygen administration during testing improves performance on some cognitive tasks [5,28], it is unknown if nighttime supplemental oxygen is effective at improving daytime cognitive performance and mood. Another possible treatment is adaptive servoventilation (ASV), which uses positive airway pressure to stabilize breathing and prevent arousals. While ASV may improve sleep quality, it is not as effective as supplemental oxygen at improving night-time saturation in the context of high altitude periodic breathing [26]. ASV therefore provides a unique opportunity to study the effects of high-altitude hypoxia separate from sleep disturbance.

We investigated whether supplemental oxygen and/or ASV sleep treatments improved daytime cognition and mood at high altitude. Subjects were tested over three consecutive days at high altitude, allowing us to also determine if cognitive function improved over time with acclimatization. We hypothesized that (1) both ASV and supplemental oxygen treatments would improve daytime cognitive function and mood and (2) acclimatization would improve cognitive function and mood on Day 3 at altitude compared to Day 1.

## Materials and methods

This study was approved by the University of California, San Diego Human Research Protections Program and was conducted in accordance with Declaration of Helsinki principles, except for registration in a database. Measurements were completed at the University of California, San Diego School of Medicine (108 m above sea level) and at Barcroft Station at the White Mountain Research Center (3,800 m above sea level). All subjects signed informed consent documents prior to participation in the study.

### Subjects

Out of 20 participants enrolled, two were excluded because they were only able to complete the supplemental oxygen treatment. Eighteen subjects completed at least two treatments and were included in the analysis (12 men, 6 women). Subject ages were 28 ± 4.2 years and BMI values were 24.1 ± 3.5 and 24.5 ± 2.2 kg/m^2^ for women and men, respectively. All subjects completed a bachelor’s degree or higher and were fluent in English. Exclusion criteria included age greater than 65 years or less than 18 years, any history of cardiovascular or pulmonary disease, smoking, obstructive or central sleep apnea at sea level, high altitude pulmonary or cerebral edema, travel above 2,500 m or sleep above 1,800 m within one month before the study, any chance of pregnancy, or use of any medications (e.g., ibuprofen) that might interfere with ventilatory control and acclimatization to high altitude [29].

All subjects completed one sea-level sleep study, as well as three consecutive sleep studies at Barcroft Station. Subjects were transported by car from sea level to Barcroft Station in three separate, non-overlapping groups. Participants slept in groups of seven or fewer individuals in a single large dormitory room in individual beds and were provided with three meals per day. This arrangement was necessary due to space limitations and ensured that all subjects slept in the same environment each night. Subjects were asked to abstain from nonsteroidal anti-inflammatory drugs (NSAIDs), alcohol, or other sedating medications during the study. Caffeine consumption was permitted only after completion of morning cognitive tests and questionnaires until 12:00 pm each day. During the day, subjects were permitted to participate in activities *ad libitum*, including moderate exercise, but they did not descend from altitude. Subjects were instructed to adhere to their normal sleep schedule. Two subjects had prior experience taking one or more of the cognitive function tests.

### Sleep treatments and polysomnography

At altitude, all subjects completed the following sleep treatments on one of three nights, in random orders: no treatment, supplemental oxygen, and ASV. The six possible treatment orders were originally distributed equally. The final dataset includes three subjects in each treatment order with the exception of 4 individuals in the ASV—No Treatment–Oxygen treatment order and 2 in the Oxygen–No Treatment—ASV order as a result of the two excluded participants. Due to the nature of the ASV device (positive airway pressure), participant blinding to treatment was not possible. On the no-treatment night, subjects slept with only polysomnography (described below). On the supplemental oxygen night, a NewLife AirSep oxygen concentrator (Chart Industries, Inc., Buffalo, NY) provided a flow rate of 2 liters per minute or higher via nasal cannula as needed to maintain awake seated saturation >95%. On the ASV night, subjects wore nasal pillows or a face mask which were individually fitted prior to bed. During the fitting, subjects acclimated to the device for 20 minutes. A ResMed S9 VPAP Adapt (ResMed, San Diego, CA) was set to expiratory positive airway pressure (EPAP) 4 cm and pressure support (PS) 3–10 cm water and settings were not adjusted during the night. These settings were decided based on clinical experience with settings that are often effective for treating central sleep apnea in non-obese individuals and tolerable for treatment-naïve individuals. No treatment was provided during the sea-level control night.

Subjects were instrumented each night with a limited channel polysomnogram (Respironics Alice PDx, Murraysville, PA) consisting of nasal pressure (except during ASV use), finger pulse oximetry, thoracic and abdominal effort bands, snore sensor, electro-oculogram, 2-channel (C3 and C4) electroencephalogram, and chin electromyogram. Each subject was also fitted with a WatchPAT device, consisting of fingertip peripheral arterial tonometry and pulse oximetry (Itamar Medical, Caesarea, Israel). Studies were scored by a Registered Polysomnographic Technologist using American Academy of Sleep Medicine criteria for staging and Chicago Criteria for events. Complete sleep results in these subjects were reported by Orr et al. (2018) [26].

### Cognitive function and mood

Subjects performed a cognitive test battery and completed mood and sleep quality questionnaires immediately after waking up and removing their instrumentation each morning. Each test item is described in detail below. Written and digital cognitive function tests were administered in the same quiet room each day, and tests were administered by the same experimenter. Subjects completed either the digital or written tests first (in the same order each day) followed by the questionnaires. Digital tests included instructions within the applications while the experimenter provided verbal instructions to subjects for written tests. To complete mood and sleep quality questionnaires, subjects were provided with a sealed folder with their ID number and paper documents inside. They were permitted to leave the room to complete these questionnaires, without discussing their responses with others, and promptly returned their documents to the experimenter. The test battery and questionnaire completion took approximately one hour. Test items were chosen based on similar batteries performed in previous high-altitude studies [30–32]. Several tests from the *Cognition* computerized test battery, which was developed to evaluate cognitive performance during spaceflight [31,33], were utilized (described below).

#### Questionnaires

The *Lake Louise AMS Score* provides a standardized score based on subject responses to questions rating physiological discomfort to high altitude [24]. Headache, gastrointestinal symptoms, fatigue and/or weakness, dizziness and/or lightheadedness, and difficulty sleeping are each scored on a scale of 0 (absent) to 3 (severe, incapacitating). Scores for each item are added to determine the final AMS score. AMS scores were recorded each evening based on the 1993 Lake Louise AMS scoring criterion while subjects were instrumented with polysomnography equipment. Participants verbally answered each question based on their level of discomfort at the time of questioning.

The *Pittsburgh Sleep Quality Index (PSQI)* was administered only on the first day of testing to obtain a baseline of each subject’s typical sleep quality [34]. The PSQI is a validated questionnaire that measures a subject’s average sleep quality over the past 30 days based on the number of times they wake up during the night, total time in bed vs total time asleep, and other factors to calculate a score [33].

The *Stanford Sleepiness Scale (SSS)* was completed each morning and night. It is an introspective measure of sleepiness in which the subjects rate their degree of sleepiness on a 7 point Likert-type scale (1 = active, vital, alert, or wide awake; 7 = No longer fighting sleep, sleep onset soon, having dream-like thoughts) throughout each hour of wakefulness during the day [35].

After completing the cognitive test battery, subjects completed a *Subjective Effort Scale* that asked four questions relating to their perceived level of effort and difficulty breathing. These included rating (1) how hard you worked to accomplish the tasks (extremely hard/not at all hard); (2) how much effort you put into concentrating on the tasks (extreme amount/none); (3) how well you performed overall on the tasks you just completed (extremely well/complete failure); and (4) any discomfort caused by your urge to breathe (extreme/none at all). Subjects provided their responses by circling a location on a 100 mm, continuous, word-labeled scale.

The 24-item *Profile of Mood States–Adolescent (POMS-A)* mood scale was chosen as it is shorter than the standard 48-item POMS questionnaire but provides comparable results [36] and is validated for use in adults [37]. Subjects were prompted with 24 words describing a feeling or emotion (panicky, lively, confused, etc.) and were asked to describe if they were currently feeling each emotion on a scale of 0 (not at all) to 4 (extremely). The 24 words on the questionnaire represent six dimensions of mood (anger, confusion, depression, fatigue, tension, and vigor). The total score for each of these six items was calculated as the sum of the responses to four mood words within its category. To calculate total mood disturbance (TMD), the total score for vigor was subtracted from the sum of total scores for tension, depression, anger, fatigue, and confusion [38].

#### Written tests

The *Trail Making Test (TMT)* evaluates visual search and motor speed skills [39]. In TMT A, subjects connected a series of 25 randomly placed numbers on a page, in order, as quickly and accurately as possible without lifting the writing instrument from the paper. TMT B also tested cognitive flexibility and executive control by requiring subjects to alternate connections from number to letter in sequential order (e.g. 1-A-2-B-C…). Time to completion and number of mistakes were recorded individually for each part. Each daily TMT included a “trial-run” of an abbreviated TMT form prior to the measured test. The potential for learning is inherent for trail making tests when taken in short intervals [39,40]. We prevented learning by implementing alternate forms of TMT A and B on each day as tested by Wagner et al. [41].

The *Digit Span Task* measures verbal short-term memory and attention [42]. We used the forward test in which the experimenter read a series of random numbers (0 to 9) to the subject, and subjects were asked to repeat the numbers in the correct order. The task began with a set of three number series containing two numbers each. If the subject repeated at least two of the three number series correctly, they advance to the next level, which contains three sets of three numbers each. Subjects continue advancing to larger number sets as they correctly complete the previous set. When the subject misses two trials within a set, the test is terminated, and the span is determined as the length of the previous correctly repeated set. While learning on this task has been demonstrated when the same number list are administered multiple times [43], to our knowledge there is no evidence of learning on this task when new number orders are presented at each test.

The *Corsi Block-Tapping Task* measures visuospatial short-term memory [44]. We used the forward test in which the subject repeated the actions of the experimenter by physically indicating (tapping) blocks (nine blocks arranged in a 3 x 3 square) in a random order. The test terminates when the subject is no longer able to recall correctly at least two out of three trials at a certain span length. This span is the value used in our analyses. Learning effects in this task have been shown to occur when the same paths are used, but these effects do not transfer to new paths after a 24-hour delay [45]. Therefore, we do not suspect learning contributed to scores on this task.

The *Verbal Paired Associates (VPA)* measures long-term memory consolidation. At night, subjects listened to a recording of a set of 20 word pairs repeated four times in different orders. Subjects were then immediately given a paper with the first word of each pair listed and were asked to recall the second word for each pair. The number of pairs recalled correctly immediately after listening to the recording was scored (immediate recall). In the morning, they were provided with the same list of words and asked to again complete the list with all the pairs they remembered. The number of pairs remembered in the morning was divided by the number of pairs remembered in the evening to obtain the percent of word pairs remembered overnight. We evaluated both immediate recall and the percent of words memorized overnight. Each night a new set of 20 word pairs was provided. Word pairs were derived at random from Payne’s semantically unrelated word pairs lists [46].

#### Digital tests

The *Emotion Recognition Test (ERT)*, which is part of the *Cognition* test package, measures facial cue emotion recognition. An iPad displayed a series of 30 portraits of people enacting emotions. Subjects chose one of five emotions to attribute to the face: happy, sad, anger, fear, or no emotion. The number of correct responses for each emotion, the false positive rate for each emotion, and the reaction time were recorded and analyzed. Learning effects are not possible in this test since subjects are never provided with the correct answers and portraits are presented in a random order in each trial.

The *Balloon Analog Risk Task (BART)* represents a measure of the propensity for risk taking [47] and is part of the *Cognition* test package. An iPad displayed a digital balloon worth $1 on the screen with two options: collect the money or pump the balloon. If subjects pump the balloon, they are awarded one additional dollar and the balloon inflates, but they risk popping the balloon and losing the money. If subjects press collect, they are awarded the value of the current balloon. We analyzed the number of pumps, the amount of money collected, and reaction times. The task consists of 30 trails while the number of pumps on which the balloon pops is random. White et al. [48] concluded that there were no significant differences between groups exposed to the BART two times or more than two times, inferring that learning did not affect performance during repeated measures of the BART.

The *Attentional Network Test (ANT)* measured the efficiency of three different attentional networks: alerting, orienting, and executive control [49]. We utilized the ANT produced by Jin Fan (Java) available through the Sackler Institute for Developmental Psychobiology (https://sacklerinstitute.org/cornell/assays_and_tools/). On a computer screen, subjects directed their attention to a cross in the center of the screen. An arrow pointing to the left or right is presented, sometimes flanked by two additional arrows on either side pointing in the same direction (congruent trials) or opposite directions (incongruent trials). The arrows are occasionally preceded by an asterisk that flashes briefly on the screen to indicate the arrows are about to be revealed (cued trials). The asterisk either flashes in the center of the screen (central cue), simultaneously above and below the central cross (double cue), or above the cross (special cue). The subject was instructed to indicate, as quickly and accurately as possible, the direction of the central arrow using a mouse when it appeared. We evaluated scores for alert effect (reaction time (RT) for no cue–RT for double cue trials), orienting effect (RT for central cue–RT for special cue trials), and conflict effect (RT for incongruent–RT for congruent trials). This version of the ANT has been shown to produce learning effects when performed in the same subject over consecutive days in executive networks but not in alerting or orienting networks [50].

We utilized the *Psychomotor Vigilance Test (PVT)*, part of the *Cognition* test package, to measure sustained attention, reflex speed, and behavioral alertness [40,51]. The subject was asked to monitor a red box in the center of an iPad screen. The box changed colors and a millisecond counter became visible, at which point subjects were instructed to tap the screen as fast as possible. We evaluated mean reaction times and errors (false starts and lapses).

### Statistical analyses

Study data were collected and managed using REDCap electronic data capture tools hosted at the University of California, San Diego [52]. We used two-way mixed linear models with repeated measures via the “lme4” package in R Studio (R Studio, Inc.) to investigate effects of day at altitude (1, 2, 3; day 0 being day of arrival) and treatment (no treatment, ASV, oxygen) on cognitive function test and mood scores. Two-way analysis of variance (type III sum of squares) tests were run on these models and post-hoc Tukey tests were used to determine significant differences across days and/or treatments when models indicated a significant effect of treatment or day at altitude on a measurement. Our dataset contains missing values for one subject who did not complete the “no-treatment” night at high altitude. Paired t-tests were completed on the remaining 17 subjects with complete sea-level and “no-treatment” high-altitude data to determine the effects of altitude itself on test scores. P-value corrections for multiple comparisons were not used due to the study’s limited sample size [53]. All statistical tests were performed in R Studio. Throughout the text, values are provided as means ± standard deviations.

## Results

Detailed sleep quality measures in these subjects as a function of day at altitude and sleep treatment have been described by Orr et al. [26]. Generally, at high altitude on “no treatment” nights, sleep efficiency was lower (high altitude (HA):0.82 ± 0.1; sea level (SL): 0.89 ± 0.07; p < 0.05) and the oxygen desaturation index was significantly higher (SL: 1.24 ± 0.90 events/h; HA: 15.56 ± 11.09 events/h; p < 0.001) than sea level values. However, arousal index did not differ across sea level and high altitude (SL: 12.4 ± 4.6; HA: 12.9 ± 4.6) but decreased from Day 1 to 2 at high altitude (Day 1: 12.8 ± 1.5; Day 2: 8.8 ± 0.9, p < 0.05). Mean saturation was lower on all days at altitude compared to sea-level measures (p < 0.001 for all days at altitude versus sea level), but did not change significantly over three days at altitude (SL: 95.5 ± 1.1;% Day 1: 89.9 ± 3.3%; Day 2: 90.5 ± 3.8%; Day 3: 91.0 ± 2.8%).

### Self-reported questionnaires

**Fig 1:**
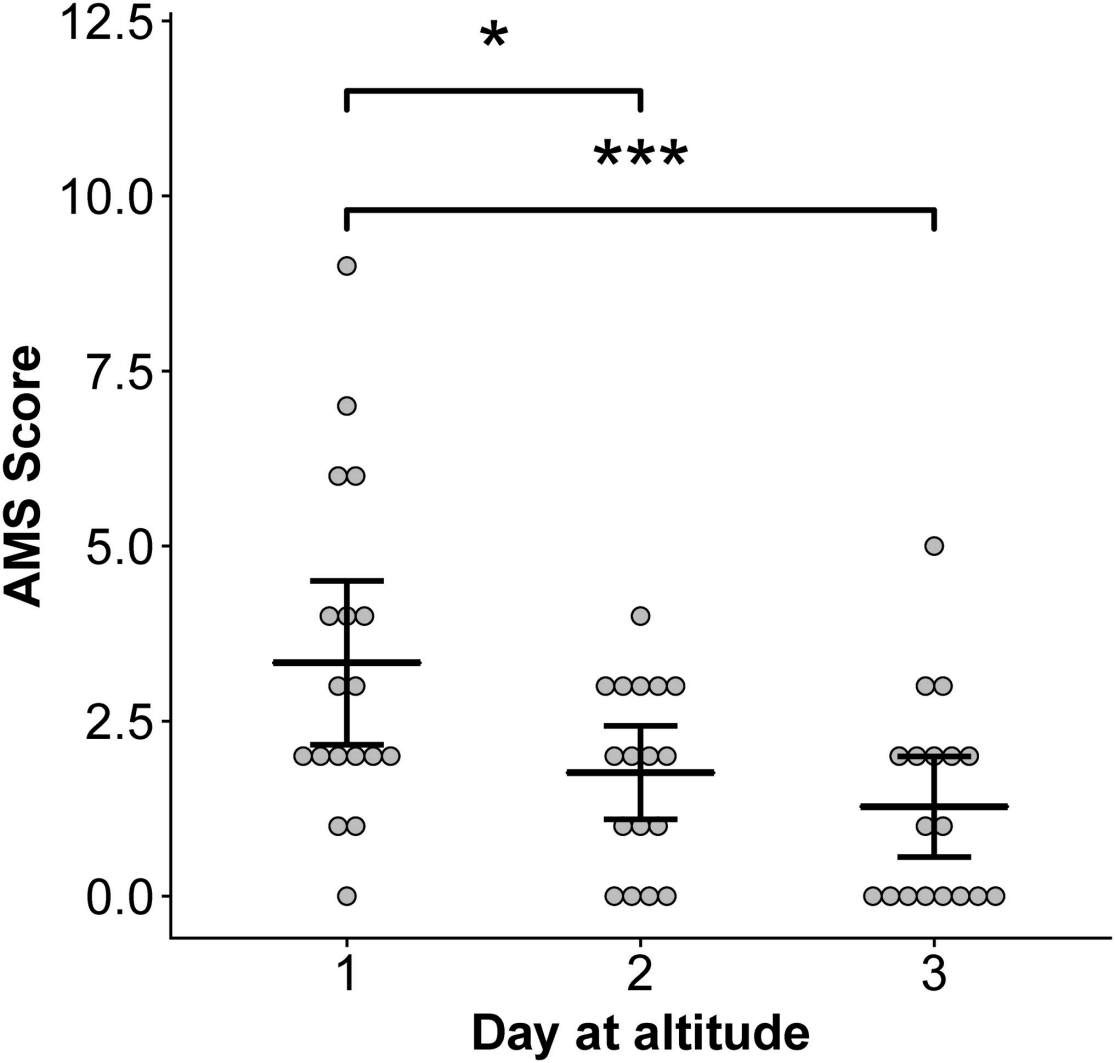
AMS scores decrease over three days at high-altitude. Points indicate daily AMS scores for each individual. Means and 95% confidence intervals are provided for each day. Asterisks indicate significant differences across days at p < 0.05 (*) and < 0.001 (***). A score of 3–5 indicates mild AMS and ≥ 6 indicates severe AMS.

There was a significant effect of day at altitude (p < 0.001) but not sleep treatment on AMS scores (Fig 1). AMS scores were highest after the first night at altitude (Day 1) and decreased significantly over the subsequent two days due to decreased severity of headache, fatigue, and gastrointestinal symptoms from Day 1 to 3. Poor sleep quality persisted throughout the study and on Day 3, 12 out of 18 subjects reported that they slept poorly (4 did not sleep as well as usual, 7 woke many times and had poor sleep, and 1 could not sleep at all).

The mean PSQI score for the cohort at the beginning of the study was 5 ± 2.6. Scores on the SSS taken at night before bed, or in the morning, were not significantly different across days or treatments at high altitude or across high-altitude and baseline nights (p > 0.05 for all comparisons).

The subjective effort scale demonstrated an effect of day on the answer to question 3, “Rate how well you performed overall on the tasks you just completed” (p < 0.02). Subjects reported that they performed the best on Day 1 at high altitude (Day 1: 13.44 ± 1.97; Day 2: 11.44 ± 3.42; Day 3: 11.39 ± 4.55). Mean responses on this question decreased each day in the ASV and oxygen treatment groups, but increased in the no-treatment group from Day 2 to 3 (NT Day 1: 12.3 ± 12.9, NT Day 2: 9.9 ± 10.5, NT Day 3: 15.0 ± 12.8), resulting in a significant interaction effect between day and treatment (p < 0.005). There was also a significant effect of day on the scores for question 4, “Rate any discomfort caused by your urge to breathe: extreme/none at all” (p < 0.05), and a significant interaction between day and treatment (p < 0.01). Subjects reported the highest discomfort breathing on Day 1 and the lowest on Day 3 (Day 1: 3.32 ± 4.92; Day 2: 2.70 ± 3.19; Day 3: 1.19 ± 0.81). Mean responses to this question decreased each day in the oxygen and no-treatment groups, but increased in the ASV group on day 2 compared to Days 1 and 3 (ASV Day 1: 1.1 ± 2.5, ASV Day 2: 5.7 ± 4.7, ASV Day 3: 1.3 ± 1.5). Subjects reported that they performed better on the tests (question 3) at high altitude compared to sea level (HA: 12.59 ± 3.13, SL: 10.47 ± 3.42; p < 0.05).

**Fig 2:**
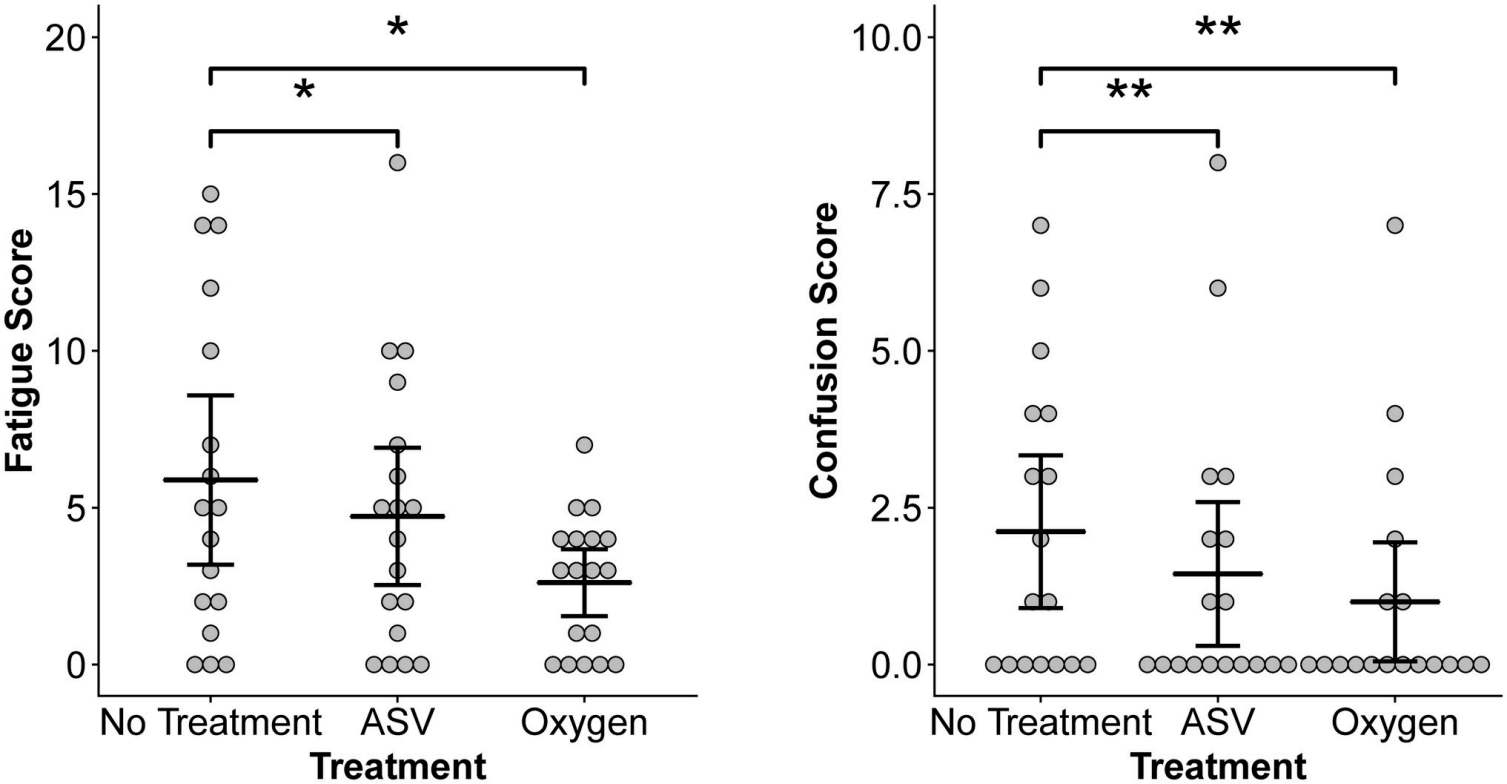
ASV and supplemental oxygen sleep treatments decrease fatigue and confusion. Results of post-hoc Tukey HSD tests on two-way ANOVA tests examining independent effects of sleep treatment on fatigue and confusion within subjects. Means and 95% confidence intervals for each treatment group are provided. Asterisks indicate significance at p < 0.05 (*) and p < 0.01 (**).

The POMS-A questionnaire revealed significant effects of treatment on self-reported levels of confusion and fatigue (Table 1; Fig 2). Subjects also reported significantly higher rates of confusion at high altitude compared to sea level (Table 1; SL: 0.54 ± 0.56, HA: 2.11 ± 2.37). Nonsignificant trends were also observed for higher fatigue and total mood disturbance at high altitude compared to sea level (Table 1). The results of post-hoc pairwise comparisons for mood states with significant effects of sleep treatment are shown in Fig 2. Confusion and fatigue levels were significantly lower following ASV and oxygen treatment nights compared to no-treatment nights. Mood did not improve with acclimatization since there were no effects of day at altitude on these mood states.

**Table 1:** Effects of altitude and sleep treatment on mood states described by POMS-A.

	Day Effects		Treatment Effects		Day x Treatment		SL v HA	
Mood	*F*	*p*	*F*	*p*	*F*	*p*	*t*	*p*
Tension	1.200	0.315	0.145	0.866	1.377	0.259	1.629	0.123
Depression	0.151	0.861	1.157	0.331	0.522	0.720	1.618	0.125
Anger	0.029	0.871	1.065	0.359	1.246	0.307	1.153	0.262
Fatigue	0.674	0.518	3.852	0.033*	2.548	0.053	2.101	0.052
Confusion	0.848	0.440	3.392	0.049*	2.518	0.059	3.615	0.002**
Vigor	2.288	0.120	0.505	0.609	1.397	0.253	0.416	0.683
TMD	1.074	0.356	2.374	0.112	1.823	0.142	1.980	0.063

Significance of day at altitude, sleep treatment, and their interaction effects on mood scores at high altitude, and significance of differences in mood states at high altitude (HA) versus sea level (SL) at p < 0.05 (*) and p < 0.01 (**) levels. TMD = Total Mood Disturbance.

### Written tests

**Fig 3:**
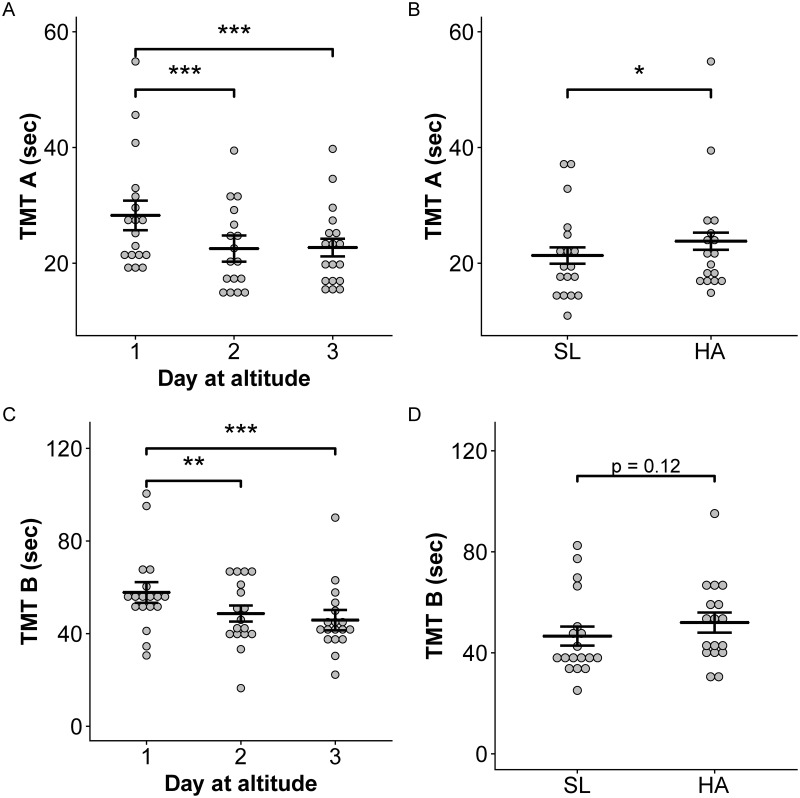
Performance on TMT A and B improved following acclimatization. Performance was worst (longer duration to complete task) on day 1 at altitude (A, C). TMT A scores were better at sea level than high altitude (B) but TMT B scores were not significantly impaired after ascent to high altitude (D). Means and 95% confidence intervals are provided. Asterisks indicate significant differences across groups at p < 0.05 (*), p < 0.01 (**), and p< 0.001 (***). SL = sea level, HA = high altitude.

The time required to complete both TMT versions A and B was highest on Day 1 at high altitude but improved over the subsequent two days (p < 0.001) (Fig 3A and 3C). There were no effects of sleep treatment on TMT A or B, and subjects performed better on the TMT A at sea level than at high altitude (Fig 3B); this pattern was similar with TMT B but not significant (Fig 3D). There were no significant differences in the number of mistakes made on TMT A or B across days at altitude or compared to their sea-level scores. Scores on the digit span and Corsi block tasks did not change significantly over days at altitude, by sleep treatments, or at high altitude compared to sea level.

The number of words memorized immediately on the VPA test was greatest on Day 2 at altitude (Day 1: 11.3 ± 5.42, Day 2: 15.05 ± 4.65, Day 3: 12.53 ± 4.46, p < 0.005). Subjects memorized more words at high altitude compared to sea level (HA: 13.0 ± 4.53, SL: 9.05 ± 5.90; p < 0.006). However, there was no effect of day or treatment on the percent of these words the subjects could recall on the following morning.

### Digital tests

**Fig 4:**
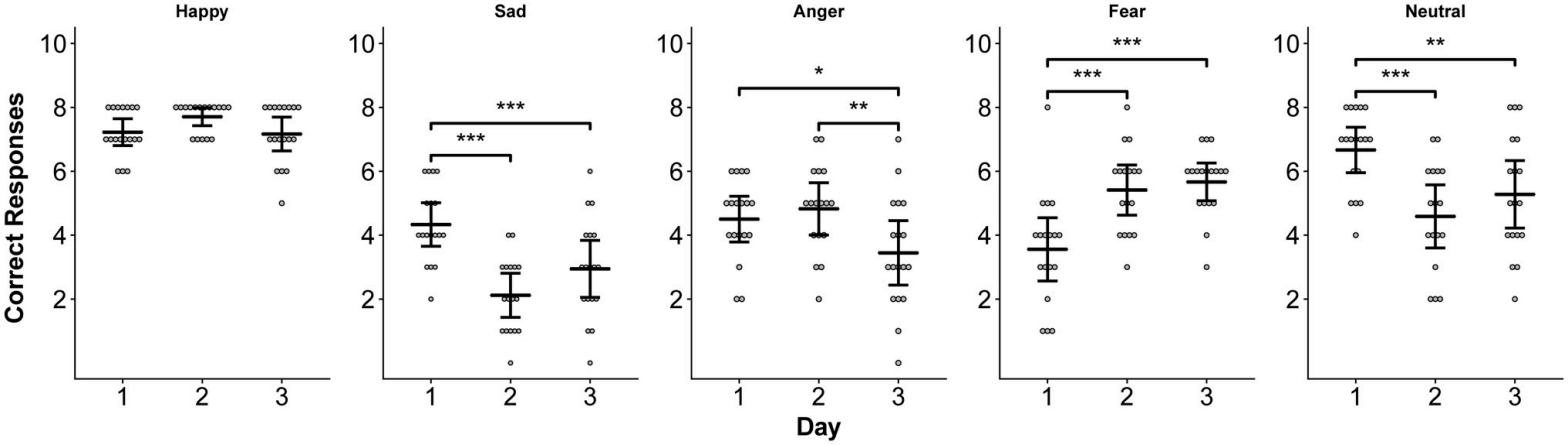
Altitude effects on emotion recognition. The total number of correctly identified expressions over three days at high altitude. Subjects were presented with happy, sad, angry, fearful, and neutral expressions. Means and 95% confidence intervals for each day are presented. Asterisks indicate significant differences across days at p < 0.05 (*), p < 0.01 (**), and p < 0.001 (***).

There was a significant effect of day at altitude, but not treatment, on the total number of correct responses on the ERT (Day 1: 26.28 ± 2.70, Day 2: 24.50 ± 2.12, Day 3: 24.50 ±3.24; p < 0.05) and subjects provided more correct answers on the ERT at sea level compared to high altitude (SL: 26.22 ± 3.00, HA: 24.82 ± 2.70; p < 0.03). There were also significant effects of day, but not treatment, on the ability to correctly identify sadness, anger, fear, and neutral expressions (Fig 4). Happiness was correctly identified more frequently at sea level than at high altitude (SL: 7.72 ± 0.57, HA: 7.12 ± 0.93; p < 0.01). There were no significant differences in the mean or median reaction times for answering on the ERT as a function of day at altitude or sleep treatment. The false positive response rate for happiness (participant indicating the person in the image was displaying happiness when they were not) was significantly higher on Days 2 and 3 at altitude compared to Day 1 (Day 1: 0.11 ± 0.32, 2: 1.06 ± 1.30, 3: 1.17 ±1.62; p < 0.003). Subjects also reported significantly higher false positive neutral responses (participant indicating the person in the image was displaying a neutral emotion when they were not) at high altitude compared to sea level (HA: 7.47 ± 3.71, SL: 5.50 ± 2.79, p < 0.04).

**Fig 5:**
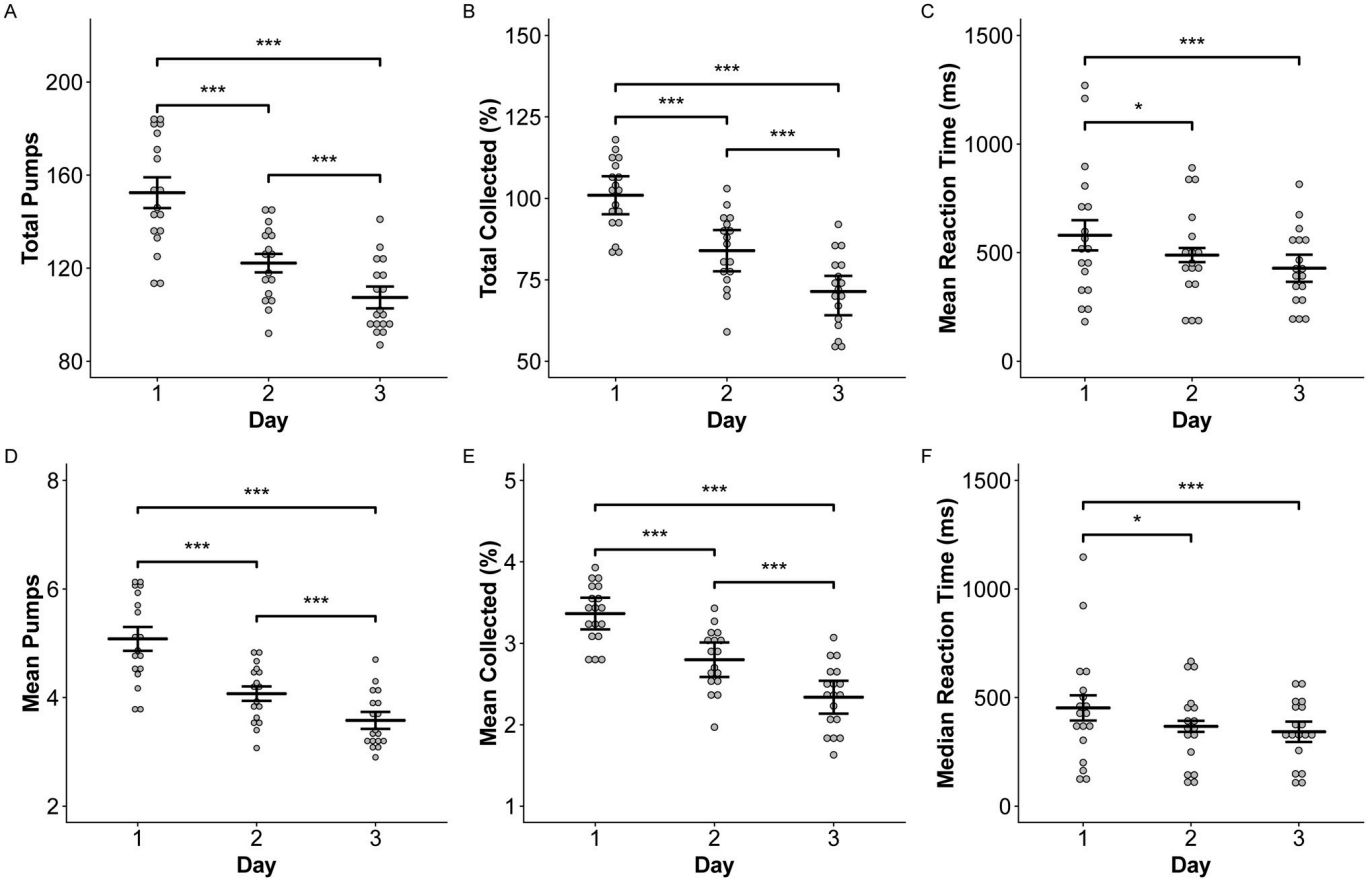
Risk-taking behavior identified by the BART decreases after acclimatization. The total number of pumps (A), mean number of pumps (D), total reward collected (B), mean reward collected (E), mean reaction time before each pump (C), and median reaction time before each pump (F) decreased over three days at high altitude. Means and 95% confidence intervals are presented. Asterisks indicate significant differences across days at p < 0.05 (*), p < 0.01 (**), and p < 0.001 (***).

In the BART test, there were significant effects of day at altitude on the total number of pumps (p < 0.0001), the mean number of pumps for each balloon (p < 0.0001), the mean reaction time before each pump (p < 0.005), the median reaction time before each pump (p = 0.006), the mean amount of money collected (p < 0.0001), and the total amount of money collected (p < 0.0001) (Fig 5). There were no significant differences in BART scores across sea-level baseline scores and no-treatment high-altitude scores, or across sleep treatments.

**Fig 6:**
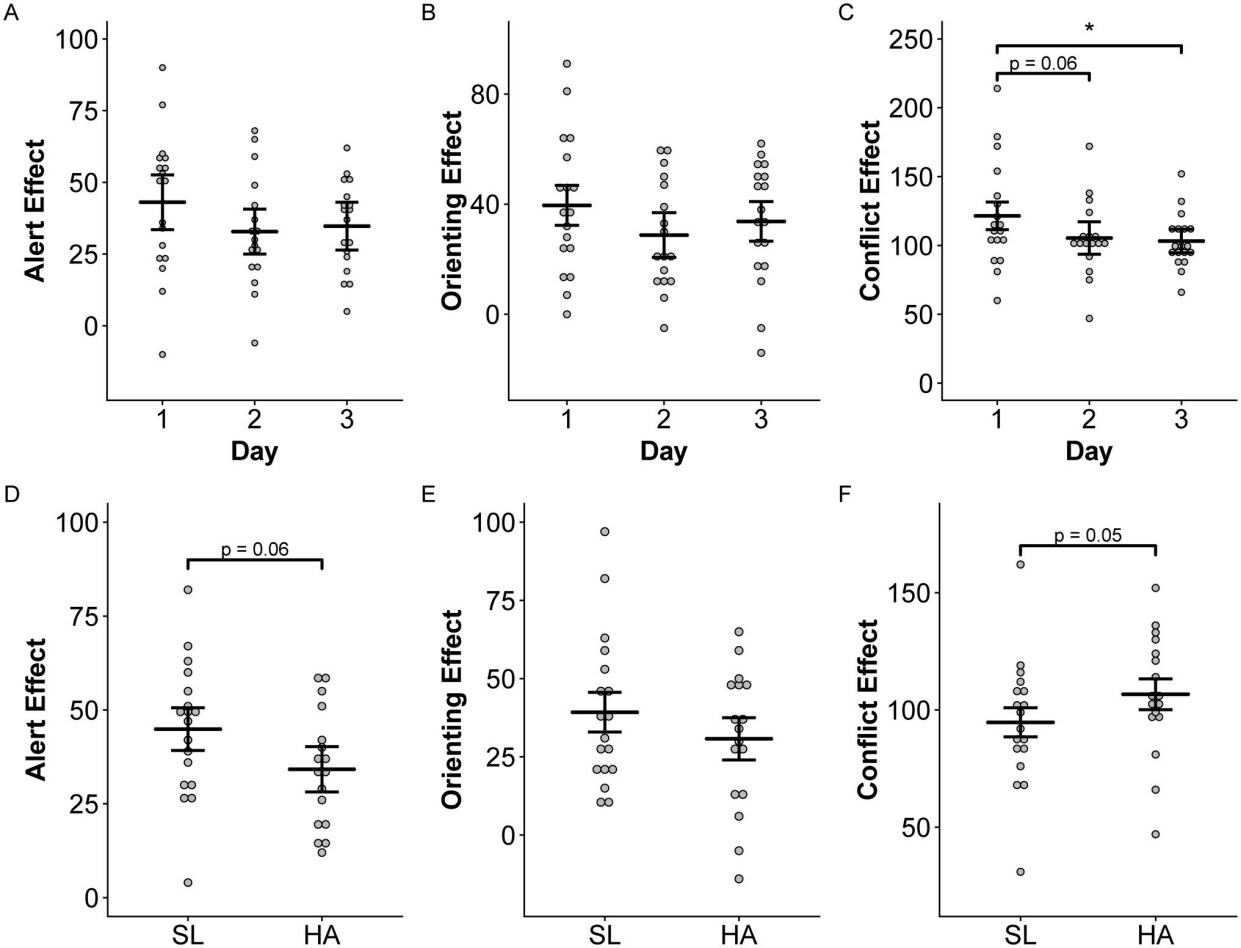
Effects of altitude on ANT performance. Alert, Orienting, and Conflict Effect scores on the ANT over three consecutive days at high altitude (top panels) and at sea level (SL) compared to the no-treatment night at high altitude (HA, bottom panels). Means and 95% confidence intervals are presented for each group. Asterisks indicate significant differences across days or locations at p < 0.05 (*).

Alert effect and orienting effect scores on the ANT were not different across days or treatments. However, there was a significant increase in conflict effect scores on Day 1 at high altitude compared to Day 3 (p < 0.05; Fig 6C), and a trend for higher conflict effect at high altitude compared to sea level (p = 0.05; Fig 6F).

**Fig 7:**
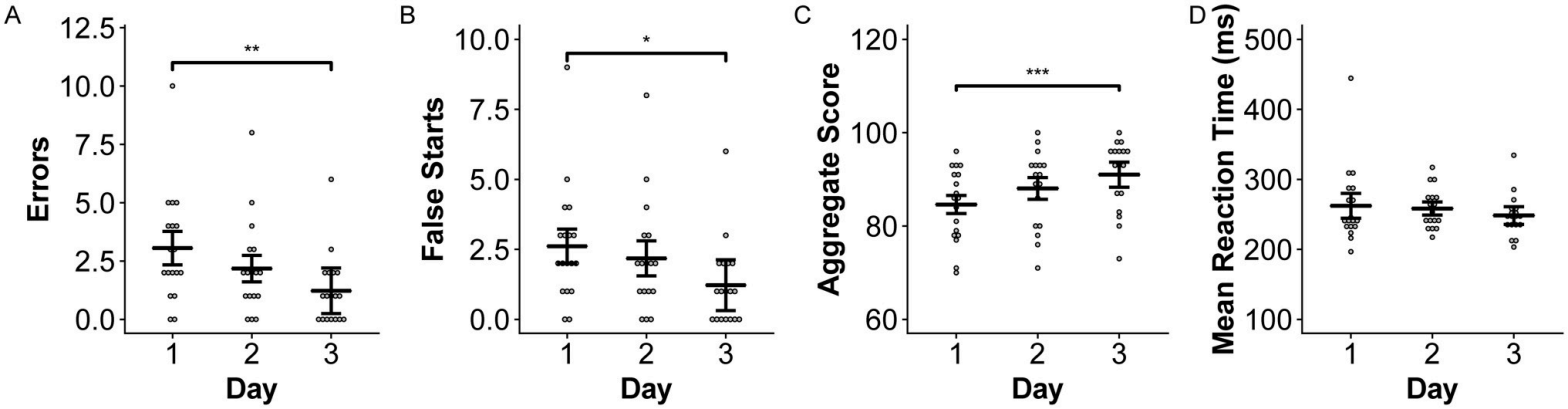
Errors and false starts on the PVT decrease following acclimatization. Errors and false starts decreased over three days at high altitude, resulting in higher aggregate scores on day 3. Mean reaction times did not decrease significantly with acclimatization. Means and 95% confidence intervals are provided for each day. Asterisks indicate significant differences across days at p < 0.05 (*), p < 0.01 (**), and p < 0.001 (***).

There was no effect of day or treatment on the mean or median reaction times on the PVT. However, there was a significant effect of day on the number of errors (Fig 7A; p < 0.01) and the number of false starts (Fig 7B; p < 0.04). Subjects made the most errors and false starts on average on Day 1 at altitude and the number of errors and false starts were both significantly different across Days 1 and 3 (Fig 7). This increase in errors and false starts reflected a decrease in the aggregate score for the PVT on Day 1 at altitude compared to Day 3 (Fig 3C; p < 0.01). There were no significant differences in any PVT scores across high altitude and sea level.

There were no significant effects of sleep efficiency or oxygen desaturation index (ODI) on any cognitive function tests when controlling for day and treatment in our sample. However, ODI had a very small negative association with the number of correct responses on the ERT (p<0.05) and the alert effect score on the ANT (p < 0.05). ODI also had a positive univariate associated with depression (p < 0.001), tension (p < 0.01), anger (p < 0.0001), fatigue (p < 0.001), and total mood disturbance (p < 0.01) scores on the mood questionnaire, but these association were lost when controlling for day and treatment effects.

## Discussion

This study evaluated if ASV and supplemental oxygen sleep treatments improve cognition and mood at high altitude. Both treatments resulted in lower levels of daytime fatigue and confusion in high-altitude visitors but did not improve any other measures of cognitive performance. While ASV did not lead to any significant improvements in sleep quality in this sample (see Orr et al. 2018 for a summary of these results) [26], the supplemental oxygen treatment significantly decreased periodic breathing and arousals and improved mean night-time saturation. The efficacy of supplemental oxygen for improving sleep quality may partially explain why the negative mood states related to fatigue and confusion were improved in nearly all subjects after supplemental oxygen use compared to the “no treatment” night (Fig 2).

It is possible the outcomes of sleep with ASV were influenced by subjects’ lack of familiarity with device, need for individual titration of settings, or intrinsic issues with the ASV algorithm in compensating for the short cycle time of periodic breathing and frequent arousals observed at high-altitude [26]. Longer acclimation periods with the ASV device may make it more effective for preventing high-altitude sleep disturbance. The efficacy may also differ in acclimatized rather than non-acclimatized individuals based on changes in control of breathing and arousals. Furthermore, ASV setting titration specific for each participant may improve ASV efficacy. Further investigation of ASV as a treatment option for high-altitude sleep disturbance is warranted because the alternatives, supplemental oxygen and acetazolamide, both have potential drawbacks. Nighttime supplemental oxygen likely slows ventilatory acclimatization, although this concept has not been demonstrated conclusively [54], and acetazolamide was recently associated with impaired memory and a reduction in processing speed and concentration 6 hours following ascent compared to matched placebo controls [55].

The decrease in AMS scores over three days at altitude (Fig 1) was primarily due to decreased headache severity, nausea, and gastrointestinal problems, while average self-reported sleep quality remained poor each of the three days. This self-reported poor sleep is supported by Orr et al. [26], which shows no improvement in oxygen desaturation index (ODI), nadir saturation, mean saturation, or arousal index in these subjects over three nights at high-altitude.

High-altitude exposure increased self-reported levels of confusion, with trends for increased fatigue and TMD, or overall negative mood (Table 1). These findings are consistent with previous studies showing increased levels of fatigue at high altitude [32,56]. There were, however, no significant changes in these mood states over three subsequent days at high altitude however, which may indicate that major contributors to fatigue and confusion were daytime hypoxia itself, or poor sleep quality, as other symptoms of AMS which may have contributed to poor mood were ameliorated over that time. The improvement in self-reported levels of fatigue and confusion after use of either ASV or supplemental oxygen sleep treatments also point to sleep quality as a potential contributor (Fig 2).

The minimal impacts of high-altitude and sleep quality on memory were surprising given short-term memory impairment has been associated with both sleep apnea and hypoxemia [57]. No differences were observed across days or treatments on the digit span and Corsi block tests. There were also no differences in the TMT A or B across sleep treatments. However, altitude impaired performance on the TMT A and performance on versions A and B improved throughout acclimatization as AMS symptoms improved. Issa et al. (2016) also show a trend for lower TMT-A performance on days when AMS symptoms were most severe [58]. Our TMT-A results support other studies showing decreased TMT performance as altitude increases [59], although due to effects of acclimatization, the speed of ascent likely plays a role in impairment severity [60].

The impacts of high altitude on emotion recognition and risk-taking behavior are notable. Subjects were significantly better at identifying happiness at sea level based on facial expressions of others. Furthermore, on Day 1 at high altitude, subjects more frequently identified sad and neutral expressions correctly but were less able to identify fear. The BART test revealed that subjects became less risky over three days at high altitude (Fig 5). On day 1, when AMS scores were highest, subjects collected higher rewards due to increased numbers of balloon pumps. Reaction times between each pump were higher on Day 1, indicating that subjects may have spent more time considering each action despite taking the riskier option (pumping the balloon) more frequently. Previous studies show that increased risk-taking behavior on the BART occurs only after 75 hours of sleep deprivation, so it is unlikely that poor sleep quality was the primary contributor to these results [61]. To our knowledge, this is the first study to investigate emotion recognition and risk-taking behavior at high altitude, although hypoxia itself has been associated with increased risk-taking behavior, especially regarding decisions involving loss [62–64].; It remains to be determined if these changes are typical at altitude and what the underlying cognitive mechanisms may be.

High altitude was also associated with impaired executive control prior to acclimatization as demonstrated by the ANT. We found trends for lower alerting and higher conflict effect scores at high altitude compared to sea level (p < 0.06, Fig 6). Lower alerting effect scores demonstrated that subjects at altitude may not benefit as much from temporal cue warnings as they do at sea level. Our conflict effect result (Fig 6C and 6F) is consistent with previous studies demonstrating complex reaction time as one of the most sensitive functions impaired at high altitude [7]. However, improvements in conflict effect scores on Days 2 and 3 at altitude may have resulted from learning effects. Repeated ANT testing has been shown to lead to improvements in the reaction time of incongruent conditions as participants learn to ignore flanking arrows [50]. Finally, the significantly lower grand mean effects on the ANT at high altitude compared to sea level likely resulted from improvements on ANT test scores on Days 2 and 3 at high altitude since the “no treatment” night, which was used as the “high altitude” value, was randomized across these three days. PVT, which provided another measure of behavioral alertness, also improved over three days at high altitude. This was due to higher error and false start frequency on Day 1 (Fig 7). Our findings of decreased errors over days at altitude are consistent with previous evaluations of the effect of acclimatization on PVT performance [40].

In conclusion, our study demonstrates that ASV and supplemental oxygen sleep treatments improve daytime feelings of fatigue and confusion at high altitude. We observed cognitive impairments that improved over subsequent days at altitude, potentially as a function of acclimatization. We also identified additional impairments at high altitude, notably cognitive flexibility and executive control, which were not affected by sleep treatments. Nighttime supplemental oxygen appears to be more effective at improving sleep quality and treating central sleep apnea events than ASV in non-acclimatized individuals at 3,800 m [26] while also providing the same improvements in mood as observed after ASV usage. The impact of ASV and long-term supplemental oxygen use on cognitive function warrants future study, especially in a larger sample following acclimatization and at higher altitudes where cognitive impairments are more severe.
